# Diagnostic and prognostic significance of cardiovascular magnetic resonance native myocardial T1 mapping in patients with pulmonary hypertension

**DOI:** 10.1186/s12968-018-0501-8

**Published:** 2018-12-03

**Authors:** Laura C. Saunders, Chris S. Johns, Neil J. Stewart, Charlotte J. E. Oram, David A. Capener, Valentina O. Puntmann, Charlie A. Elliot, Robin C. Condliffe, David G. Kiely, Martin J. Graves, Jim M. Wild, Andy J. Swift

**Affiliations:** 1 POLARIS, Academic Radiology, Department of Infection, Immunity and Cardiovascular Disease, University of Sheffield, Sheffield, UK; 2 Division of Bioengineering and Bioinformatics, Graduate School of Information Science and Technology, Hokkaido University, Hokudai, Japan; 3 Institute for Experimental and Translational Cardio Vascular Imaging, University Hospital Frankfurt, Frankfurt, Germany; 4 Pulmonary Vascular Disease Unit, Sheffield Teaching Hospitals NHS Trust, Sheffield, UK; 5 INSIGNEO, Institute for in-silico medicine, Sheffield, UK; 6 University of Cambridge School of Clinical Medicine, Cambridge University, Cambridge, UK

**Keywords:** Pulmonary hypertension, T1 mapping, Cardiovascular magnetic resonance, MOLLI

## Abstract

**Background:**

Native T1 may be a sensitive, contrast-free, non-invasive cardiovascular magnetic resonance (CMR) marker of myocardial tissue changes in patients with pulmonary artery hypertension. However, the diagnostic and prognostic value of native T1 mapping in this patient group has not been fully explored. The aim of this work was to determine whether elevation of native T1 in myocardial tissue in pulmonary hypertension: (a) varies according to pulmonary hypertension subtype; (b) has prognostic value and (c) is associated with ventricular function and interaction.

**Methods:**

Data were retrospectively collected from a total of 490 consecutive patients during their clinical 1.5 T CMR assessment at a pulmonary hypertension referral centre in 2015. Three hundred sixty-nine patients had pulmonary hypertension [58 ± 15 years; 66% female], an additional 39 had pulmonary hypertension due to left heart disease [68 ± 13 years; 60% female], 82 patients did not have pulmonary hypertension [55 ± 18; 68% female]. Twenty five healthy subjects were also recruited [58 ±4 years); 51% female]. T1 mapping was performed with a MOdified Look-Locker Inversion Recovery (MOLLI) sequence. T1 prognostic value in patients with pulmonary arterial hypertension was assessed using multivariate Cox proportional hazards regression analysis.

**Results:**

Patients with pulmonary artery hypertension had elevated T1 in the right ventricular (RV) insertion point (pulmonary hypertension patients: T1 = 1060 ± 90 ms; No pulmonary hypertension patients: T1 = 1020 ± 80 ms *p* < 0.001; healthy subjects T1 = 940 ± 50 ms *p* < 0.001) with no significant difference between the major pulmonary hypertension subtypes. The RV insertion point was the most successful T1 region for discriminating patients with pulmonary hypertension from healthy subjects (area under the curve = 0.863) however it could not accurately discriminate between patients with and without pulmonary hypertension (area under the curve = 0.654). T1 metrics did not contribute to prediction of overall mortality (septal: *p* = 0.552; RV insertion point: *p* = 0.688; left ventricular free wall: *p* = 0.258). Systolic interventricular septal angle was a significant predictor of T1 in patients with pulmonary hypertension (*p* < 0.001).

**Conclusions:**

Elevated myocardial native T1 was found to a similar extent in pulmonary hypertension patient subgroups and is independently associated with increased interventricular septal angle. Native T1 mapping may not be of additive value in the diagnostic or prognostic evaluation of patients with pulmonary artery hypertension.

**Electronic supplementary material:**

The online version of this article (10.1186/s12968-018-0501-8) contains supplementary material, which is available to authorized users.

## Background

Myocardial tissue changes have been visualised in patients with pulmonary hypertension using cardiovascular magnetic resonance (CMR) T1 mapping, T2 mapping and late gadolinium enhancement (LGE) CMR. Native myocardial changes measured using native T1 mapping have been shown across a range of cardiac diseases in clinical subjects [1, 2]. It has been shown that in patients with pulmonary artery hypertension (PAH) the right ventricular (RV) insertion point T1 is elevated. Several studies also found elevated T1 in the interventricular septum and, in one case, the left ventricular (LV) free wall [3–7].

Patients with pulmonary hypertension have been shown to have worse prognosis in the presence of myocardial changes visualized by LGE at the RV insertion point [8] and in the septal myocardium [9], although the independent prognostic significance of the myocardial tissue changes in pulmonary hypertension remains unclear [10]. Elevated native T1 at the insertion point occurs in a similar location and pattern to LGE. The co-occurrence of LGE and elevated T1 implies that they may have common mechanistic causes. However, studies in both patients and animal models have reported higher native T1 in the RV insertion points than in controls, despite no LGE being seen in this region, which indicates that native T1 may be a more sensitive marker of myocardial changes than the presence of LGE [3, 11]. Therefore, T1 mapping may provide a contrast-free and sensitive marker of myocardial tissue changes in pulmonary artery hypertension, and there is reason to suspect that T1 changes may have prognostic relevance.

Pulmonary hypertension is a heterogeneous condition with 5 diagnostic subcategories: PAH; pulmonary hypertension due to left heart disease pulmonary hypertension due to lung disease and/or hypoxia; chronic thromboembolic pulmonary hypertension (CTEPH) and pulmonary hypertension with unclear or multifactorial causes. To date T1 mapping studies have been performed in relatively small populations, with only one report wherein diagnostic subcategories were considered separately [4] and therefore it is unclear whether myocardial T1 changes in patients with pulmonary hypertension are homogeneous across patient subtypes.

RV remodelling and failure occur as a result of prolonged elevations of RV afterload. Elevated mean pulmonary artery pressure (PAP) and pulmonary vascular resistance (PVR) are characteristic features of the condition, however it is the failure of the RV that is the key determinant of adverse outcome [9, 12–16]. Early markers of adverse cardiac remodelling may be able to guide treatment strategies, predicting therapy response and failure. It is unclear whether elevated myocardial T1 is associated with prognostic features in pulmonary hypertension, and whether it can provide additive information to patient outcome.

In this work, we sought to determine whether elevation of the native T1 in myocardial tissue in pulmonary hypertension: (a) varies according to pulmonary hypertension subtype; (b) is related to adverse outcome independent of RV size and function, and (c) is associated with biventricular remodeling and function.

## Methods

### Subjects

**Fig. 1:**
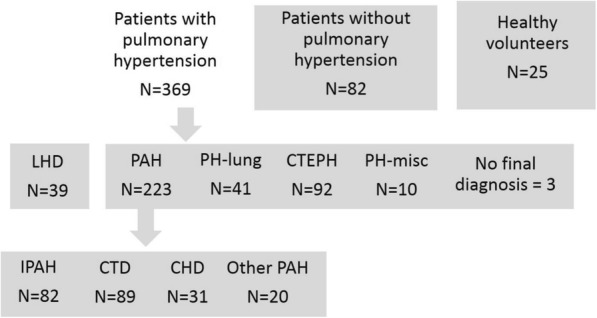
Patient flowchart. Patients were grouped for statistical analysis as indicated. Patients with pulmonary hypertension were compared to two control groups. Patients with left heart disease (LHD), pulmonary arterial hypertension (PAH), PAH due to lung disease and/or hypoxia (PH-lung), chronic thromboembolic pulmonary hypertension (CTEPH) and pulmonary hypertension with unclear or multifactorial causes (PH-misc) were compared using ANOVA. Patients with idiopathic pulmonary arterial hypertension (IPAH), connective tissue disease (CTD), congenital heart disease (CHD) and other types of PAH were compared using ANOVA. Patients with PAH were assessed for independent prognostic value of T1. Three patients had previous right heart catheter data showing mean PAP > 25 mmHg but did not have a final diagnosis, and were included in analysis of pulmonary hypertension patients

Approval for analysis of imaging data was granted by the local research ethics committee and consent was waived for this retrospective database study. Consecutive patients with suspected pulmonary hypertension underwent CMR including a modified Look-Locker inversion recovery (MOLLI) sequence as part of their clinical assessment at a tertiary pulmonary hypertension referral centre between 01.01.2015 and 31.12.2015. Exclusion criteria were applied to all subjects according to standard criteria for undergoing CMR. A total of 490 patients were identified. Three hundred sixty-nine patients had a diagnosis of pulmonary hypertension without left heart disease as specified below, 39 patients were identified with pulmonary hypertension associated with left heart disease, and 82 did not have a diagnosis of pulmonary hypertension - mean PAP < 25 mmHg – and were used as a control group, see Fig. 1. Twenty five age matched healthy subjects with no current cardiac or respiratory symptoms and no history of cardiac disorders were also recruited as an additional control group. Patients with a mean PAP ≥ 25 mmHg and pulmonary capillary wedge pressure (PCWP) > 15 mmHg were considered to have a diagnosis of pulmonary hypertension due to left heart disease, and patients with a mean PAP < 25 mmHg were considered to have a diagnosis of no pulmonary hypertension. Subtypes of pulmonary hypertension were defined as previously described [17].

The patients with pulmonary hypertension included the following sub-group diagnoses: Group 1 pulmonary arterial hypertension; Idiopathic PAH, *n* = 83; PAH heritable, *n* = 5; PAH associated with connective tissue disease (PAH-CTD), *n* = 89; PAH associated with congenital heart disease (CHD), *n* = 31; PAH associated with portal hypertension, *n* = 9; PAH drug/toxin induced, n = 3; PAH other, *n* = 3. Group 2 left heart disease, *n* = 39 (note these patients were considered separately – see Fig. 1). Group 3 PH associated with lung disease, *n* = 41. Group 4, CTEPH *n* = 92; group 5, sarcoidosis, *n* = 10; patients without diagnosis: 3.

All patients with PAH (*n* = 223) were treated with PAH therapy. Patients with PAH were strictly defined for this study as PCWP < 16 and no evidence of lung disease or left heart disease. Chronic thromboembolic pulmonary hypertension (CTEPH) patients and some aetiologies of Group 5 pulmonary hypertension were treated as appropriate. Patients with left heart disease were divided into two groups, firstly typical left heart disease (*n* = 35) and secondly, PAH with left heart disease comorbidity (n = 4). Patients in the PAH associated with lung disease were divided into severe PAH-lung disease (*n* = 13) and PAH with non-severe lung disease comorbidity (*n* = 28). A summary of all subject characteristics is shown in Table 1, and a detailed breakdown of the subgroups provided in Additional file 1: Table S1.

**Table 1:** Characteristics of subjects

N	Patients: PAH	Group 3 PH LHD	Patients: no PAH	Healthy Subjects
	369	39	82	24
Demographics and characteristics				
Age (years)	58.2 ± 15.0	68.1 ± 12.7**	55.4 ± 18.3	58.1 ± 3.88
Sex (female %)	66.0	59.5	67.1	50.9
Subgroup diagnoses (n)		N/A	N/A	–
Group 1 PAH	*n* = 223			
PH-Lung	*n* = 41			
CTEPH	*n* = 92			
PH-MISC	*n* = 10			
BSA (m^2^)	1.81 ± 250	1.89 ± 0.26	1.85 ± 0.25	
CMR				
Heart Rate (bpm)	72.17 ± 14.4	69.81 ± 3.8	67.9 ± 10.3**	
CMR at incident vs prevalent time (incident %)	*N* = 165 (45%)	*N* = 31 (80%)	*N* = 82 (100%)	
RV insertion T1 (ms)	1065 ± 86	1074 ± 10	1017 ± 69	943 ± 52**
Septal T1 (ms)	975 ± 67	986 ± 73	976 ± 75	940 ± 56*
LV free wall T1 (ms)	965 ± 68	982 ± 81	961 ± 66	913 ± 55**
RVEDV Index (ml/m^2^)	87.2 ± 34.1	81.9 ± 31.7	68.0 ± 20.4**	
RVESV Index (ml/m^2^)	51.7 ± 29.0	45.4 ± 23.1	31.1 ± 11.6**	
RVSV Index (%)	35.6 ± 14.2	36.5 ± 12.1	36.9 ± 12.9	
RVEF (%)	43.1 ± 13.6	46.8 ± 11.2	54.4 ± 10.0**	
LVEDV Index (ml/m^2^	58.5 ± 16.1	68.9 ± 21.2**	66.6 ± 16.1**	
LVESV Index ml/m^2^)	19.2 ± 8.2	26.1 ± 16.6**	21.3 ± 8.2*	
LVSV Index (%)	39.2 ± 11.9	42.8 ± 13.4	45.3 ± 10.8**	
LVEF (%)	67.3 ± 9.7	63.8 ± 14.5	68.3 ± 7.6	
Systolic septal angle (°)	165.5 ± 23.9	145.9 ± 15.5	150.5 ± 108.4*	
Diastolic septal angle (°)	143.34 ± 11.0	138.3 ± 10.3**	134.9 ± 7.9*	
RV mass index (g/m^2^)	21.9 ± 12.5	14.4 ± 5.5**	11.9 ± 5.8*	
LV mass index (g/m^2^)	48.0 ± 12.3	57.2 ± 21.1**	47.7 ± 10.7	
VMI (ratio)	0.47 ± 0.27	0.28 ± 0.13**	0.26 ± 0.18*	

*WHO* world health organisation, *PAH* ipulmonary arterial hypertension, *CTEPH* chronic thromboembolic pulmonary hypertension, *RVEDV* right ventricular end diastolic volume, *RVESV* right ventricular end systolic volume, *RVSV* right ventricular stroke volume, *RVEF* right ventricular ejection fraction, *VMI* ventricular mass index, *LVEDV* left ventricular end diastolic volume, *LVESV* left ventricular end systolic volume, *LVSV* left ventricular stroke volume, *LVEF* left ventricular ejection fraction, *VMI* ventricular mass index, *CMR* cardiovascular magnetic resonance

*P* values indicate comparisons between patients with PH and the other two groups. * signifies a *p* value < 0.05, ** signifies a *p* value < 0.025

### Right heart catheterisation

All patients underwent right heart catheterisation. Right heart catheterisation was performed by a using a balloon-tipped 7.5 Fr thermodilution catheter (Becton-Dickinson, Franklin Lakes, New Jersey, USA), typically via the internal jugular vein using a Swan-Ganz catheter. PVR index (PVRI) was defined as (mPAP-PCWP)/CI, where CI is cardiac index, measured by thermodilution technique. Patients with right heart catheterisation within 30 days of their CMR were included in analysis of associations between right heart catheterisation metrics and myocardial T1.

### CMR acquisition

CMR was performed on a whole body 1.5 T scanner (HDx scanner, GE Healthcare, Waukesha, Wisconsin, USA), using an 8-channel cardiac coil. Subjects were scanned in the supine position with electrocardiogram (ECG) gating. Short axis cine images were acquired using a multi-slice balanced steady state free precession (bSSFP) sequence with: temporal phases per cardiac cycle: 20; field of view: 480 mm; matrix: 256 × 256; bandwidth: 125KHz/pixel; TR: 3.7 ms; TE: 1.6 ms.

T1 mapping was performed using a 2D 3–3-5 MOLLI sequence in a single short axis slice [17]. A bSSFP acquisition was executed at each inversion time point with the following sequence parameters: Flip angle: 35°; image dimensions: 128 × 128; TR: 3.20 ms; TE: 1.41 ms; parallel imaging using sensitivity encoding with acceleration factor 2; FOV: 400 mm; slice thickness: 5.1 mm.

### CMR image analysis

Cine CMR analysis was performed prospectively on a GE Advantage Workstation 4.1 by a radiographer with 10 years’ experience, who was blinded to the patient’s clinical data. Right and left endocardial and epicardial surfaces were manually traced on the short axis cine images to obtain the volumetric indices; RV end diastolic volume (RVEDV), RV end systolic volume (RVESV), LV end diastolic volume (LVEDV) and LV end systolic volume (LVESV). From the end diastolic and end systolic volumes, RV ejection fraction (RVEF) and LV ejection fraction (LVEF) were calculated. The interventricular septum was included in the LV mass. RV mass was estimated from the sum total of the myocardial slice volumes and an assumed myocardial density of 1.05 g/cm^3^. Ventricular mass index was calculated as VMI = RV mass / LV mass. CMR volume and mass measurements were indexed for body surface area where appropriate. Interventricular septal angle was measured by determining the angle between the mid-point of the interventricular septum and the RV insertion points at RV end systole [18].

**Fig. 2:**
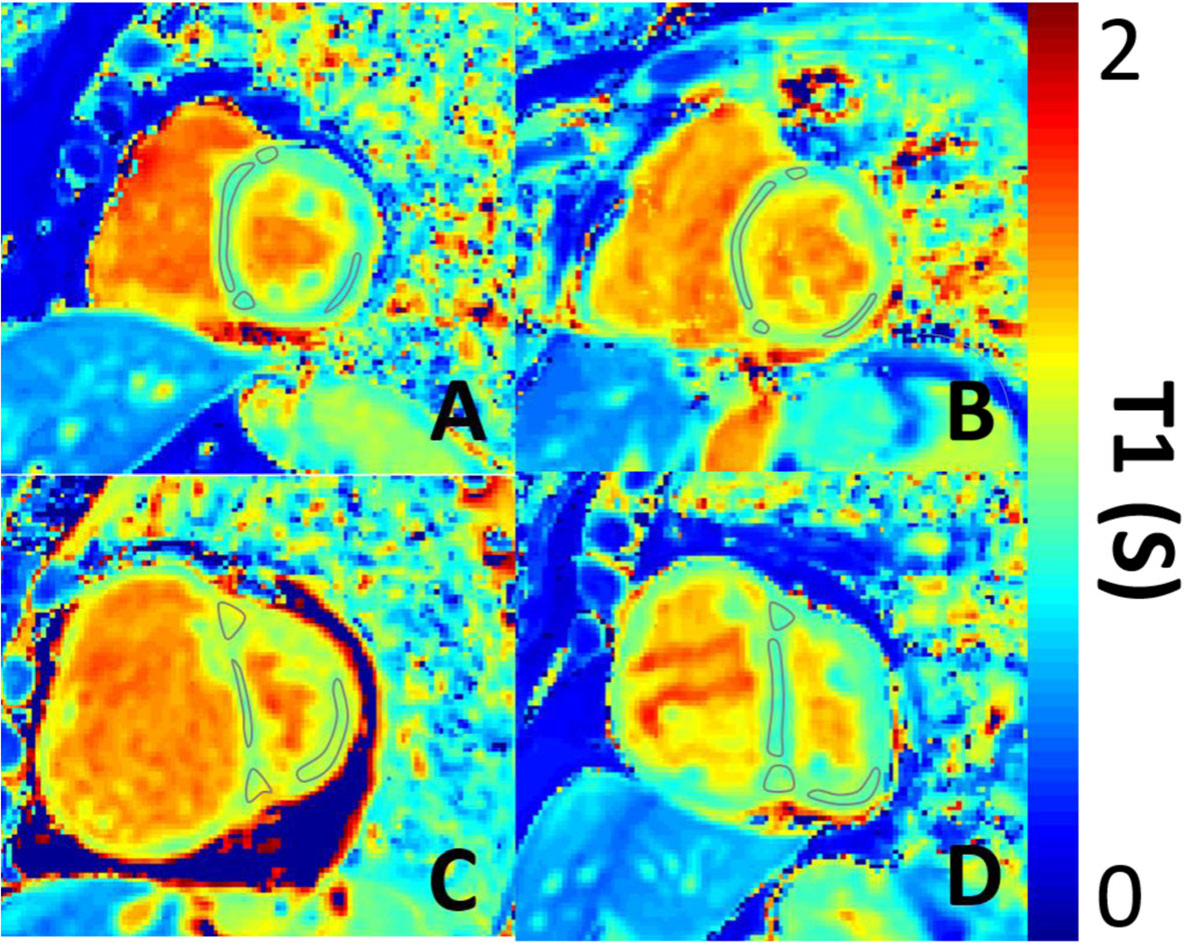
Representative T1 maps. Representative T1 maps in short axis geometry of **a**) a healthy subject, **b**) a patient without pulmonary artery hypertension, **c**) a patient with idiopathic PAH and **d**) a patient with left heart diseaes. Demonstrative regions of interest are places on the RV insertion points, interventricular septum and LV free wall

All MOLLI images were spatially registered to correct any respiratory or cardiac motion using a Matlab (Mathworks, Natick, Massachusetts, USA)-based non-rigid image registration algorithm [19], which was integrated into a Matlab voxelwise T1 mapping algorithm based on the work by Xue et al. [20]. Analysis of T1 maps was performed by a Cardiothoracic Radiologist with 7 years CMR experience, blinded to the patient’s demographic and clinical data. Regions of interest were drawn on the interventricular septum, RV insertion points and LV free wall, see Fig. 2 [21]. A measure of ΔT1_RS_ was used to assess elevation of the T1 in the RV insertion points, normalised to septal T1. Regions of interest were compared across pulmonary hypertension subtypes (see flowchart in Fig. 1). All T1 maps were analysed by both the same observer, and a second observer, who was a trained CMR researcher with 2 years of experience analysing T1 mapping images blinded to previous results and patient data for interobserver and intraobserver reproducibility.

### Follow up period

Patients with PAH were followed up with a census date of 30.01.2018 with a mean follow-up period of 27 ± 8 months and a median follow-up period of 30 months from CMR to death or census.

### Statistical analysis

Statistical analysis was performed and presented using SPSS 21 (SPSS, Statistical Package for the Social Sciences, International Business Machines, Inc., Armonk, New York, USA). Data are presented as mean ± standard deviation. Group comparisons were made using two-tailed ANOVA; post-hoc Bonferroni was used for multiple group corrections. Pearson’s correlation coefficient was used to assess the strength of correlations. Receiver operating characteristic (ROC) analysis was employed to assess the diagnostic accuracy of T1 metrics with area under the curve. Intra and interobsever variability was assessed using the intraclass correlation coefficient (ICC); absolute values and 95% confidence intervals. To control for the heterogeneity of aetiology and therapy differences between the different subtypes of pulmonary hypertension, we elected to study the prognostic value of T1 metrics in patients with PAH alone. This controls for differences in outcome caused by factors such as severe lung disease in respiratory patients and chronic thromboembolic disease in patients undergoing pulmonary endarterectomy. Log-log plots were produced for T1 metrics to assess proportional hazards, with variables dichotomised by median values, and prognostic value was assessed using univariate Cox proportional hazards regression analysis. Variable scaling (Z-score) was performed to allow direct comparison of hazard ratios of all continuous variables by subtracting the mean from individual values and dividing by the standard deviation of the variable. Groups were compared using the log-rank (Mantel-Cox) test. Unless otherwise stated, a value of *p* < 0.05 was considered statistically significant.

## Results

Characteristics of the study populations are given in Table 1. Additional data on characteristics of PAH subtypes are given in Additional file 1**:** Tables S1 and S2. Patients with PAH were not significantly different in age or sex than patients without PAH or healthy subjects. Figure 1 illustrates the classification of the patients included in the study.

### Native myocardial T1 mapping

**Fig. 3:**
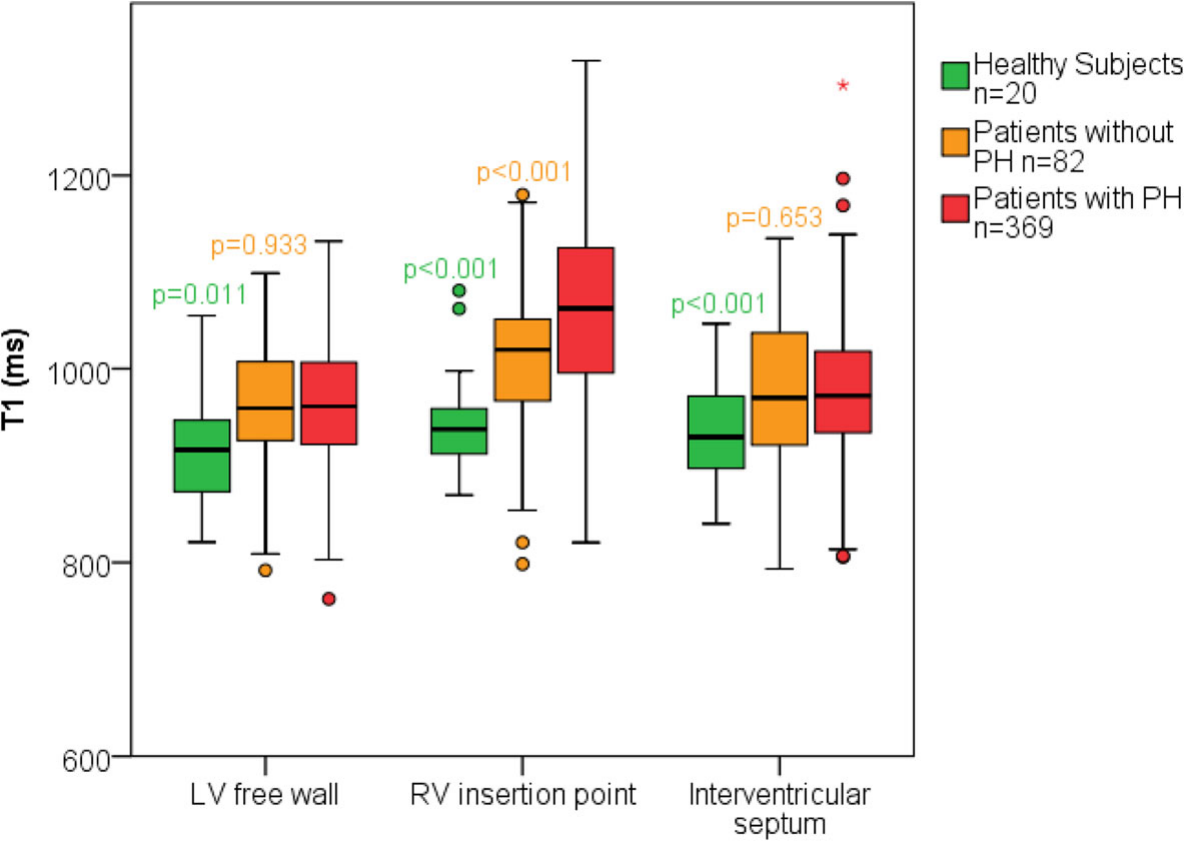
Mean T1 in patients with pulmonary artery hypertension, patients without pulmonary artery hypertension and healthy subjects. Box plot of the mean T1 for septal, RV insertion point and LV free wall regions. *P* values are given for ANOVA comparisons between patients with pulmonary artery hypertension and control groups above each respective control group. O Represents outliers * represents far outliers

In patients with PAH, T1 was significantly elevated at the RV insertion points when compared to healthy subjects and patients without PAH (patients with pulmonary hypertension, T1 = 1065 ± 86 ms; healthy subjects, T1 = 943 ± 52 ms, *p* < 0.001; patients PAH T1 = 1017 ± 69 ms, p < 0.001). Patients with PAH also had elevated T1 in the LV free wall and septum when compared to healthy subjects but not patients PAH, (patients with PAH: LV free wall T1 = 975 ± 67 ms, septal T1 = 965 ± 68 ms; patients without PAH: LV free wall T1 = 961 ± 66 ms, septal T1 = 976 ± 75 ms; healthy subjects LV free wall T1 = 913 ± 55 ms, p < 0.001, septal T1 = 940 ± 56 ms, *p* = 0.011). See Table 1 and Figs. 2, 3. Patients with PAH who were undergoing treatment did not have significantly different T1 to those who were untreated (septal: *p* = 0.182; RV insertion point: *p* = 0.977; LV free wall: *p* = 0.46). The standard deviation of T1 s within the RV insertion points was significantly higher in patients with PAH (SD = 62.2 ms) compared to healthy subjects (SD = 36.5 ms). The standard deviation of T1 s within the LV free wall and septum were not significantly different in healthy subjects when compared to patients (septal: 54.4 ms, LV free wall: 42.9 ms) with PAH (septal: 54.6 ms, LV free wall: 44.6 ms).

### Diagnostic accuracy of myocardial T1

The T1 at the RV insertion points was the most statistically significant measurement for discriminating between subjects and patients with PAH, followed by ΔT1_RS_. However, no T1 region was successful in discriminating between patients without PAH and patients with PAH, see Table 2.

**Table 2:** ROC curve analysis for distinguishing patients without PH and patients with PH

	Area under the curve	Lower 95% Confidence interval	Upper 95% Confidence interval
RV mass Index	0.807	0.760	0.855
Systolic septal angle	0.850	0.809	0.892
Septal T1	0.502	0.426	0.578
RV insertion point T1	0.654	0.587	0.720
ΔT1_RVIP-septum_	0.675	0.612	0.738
LV free wall T1	0.512	0.442	0.582

Legend: ΔT1_RVIP-septum_ = RV insertion point T1 – septal T1

### T1 changes in pulmonary artery hypertension subtypes

**Fig. 4:**
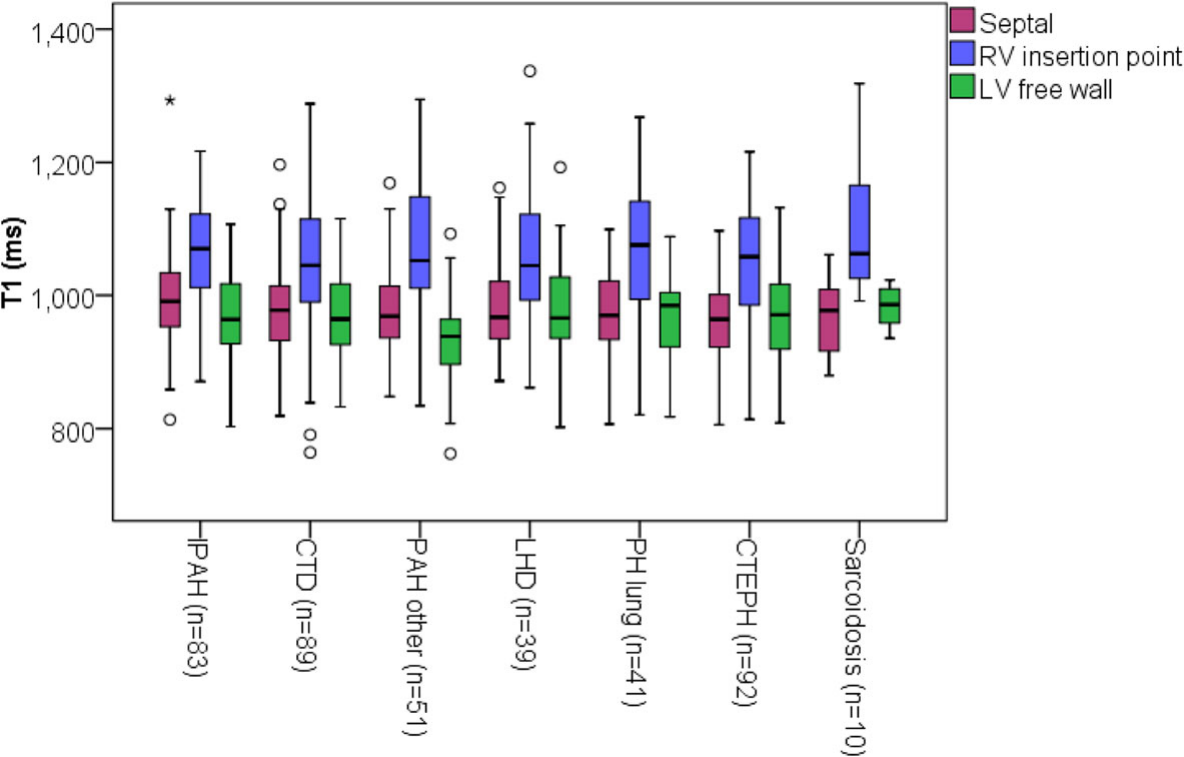
Mean T1 in subgroups of patients with pulmonary hypertension. Box plot of mean T1 for septal, RV insertion point and LV free wall regions for pulmonary artery hypertension patient subtypes. PAH other includes all patients with PAH who do not have IPAH,CTD or CHD. O Represents outliers * represents far outliers

Comparisons were made between PAH subgroups and there were no significant differences between subgroups. For PAH patients, LV free wall T1 was significantly higher (*p* = 0.046) in patients with CTD than patients with CHD. There were no other significant differences between groups, see Fig. 4.

### Relationship between T1 and other measures of cardiac structure and function

Across the population of PAH patients scanned, the RV insertion point T1 had the strongest relationship with RV function. The mean insertion point T1 correlated significantly with RVEDV index, RVESV index and RVEF. Additionally, the RV insertion point T1 showed significant correlations with RV mass index, the VMI and the systolic and diastolic septal angle, see Table 3. Septal T1 and LV free wall T1 did not correlate with any of the imaging markers of RV function or LV function. However, diastolic septal angle and septal T1 showed a positive correlation (*R* = 0.152), see Table 3.

**Table 3:** Correlations between MR parameters in septal, RV insertion point and LV free wall T1

	Septal T1 (ms)	RV insertion point T1 (ms)	LV free wall T1 (ms)
Age (years)	*r* = − 0.074 *p* = 0.159	*r* = 0.007, *p* = 1.000	*r* = − 0.086, *p* = 0.168
BSA (m2)	*r* = 0.015, *p* = 0.771	*r* = 0.041, *p* = 0.444	*r* = −0.006, *p* = 0.915
Heart Rate (bpm)	*r* = 0.011, *p* = 0.836	*r* = − 0.073, *p* = 0.168	*r* = − 0.086, *p* = 0.103
RVEDV Index (ml/m2)	*r* = 0.066, *p* = 0.213	*r* = 0.265**, *p* < 0.001**	*r* = 0.076, *p* = 0.151
RVESV Index (ml/m2)	*r* = 0.027, *p* = 0.615	*r* = 0.290, p < 0.001**	*r* = 0.088, *p* = 0.097
RVSV Index (%)	*r* = 0.103, *p* = 0.050	*r* = 0.003, *p* = 0.958	*r* = 0.044, *p* = 0.406
RVEF (%)	*r* = 0.003, *p* = 0.951	*r* = −0.238, *p* < 0.001**	*r* = −0.080, *p* = 0.129
LVEDV Index (ml/m2)	*r* = −0.035, *p* = 0.505	*r* = −0.060, *p* = 0.257	*r* = − 0.018, *p* = 0.734
LVESV Index ml/m2)	*r* = 0.005, *p* = 0.924	*r* = 0.011, *p* = 0.841	*r* = −0.051, *p* = 0.335
LVSV Index (%)	*r* = 0.051, *p* = 0.335	*r* = −0.088, *p* = 0.096	*r* = 0.011, *p* = 0.841
LVEF (%)	*r* = 0.028, *p* = 0.598	*r* = −0.066, *p* = 0.217	*r* = 0.050, *p* = 0.347
Systolic septal angle (°)	*r* = 0.096, *p* = 0.069	*r* = 0.238, *p* < 0.001**	*R* = 0.017, *p* = 0.742
Diastolic septal angle (°)	*r* = 0.157, *p* = 0.003**	*r* = 0.253, *p* < 0.001**	*r* = 0.017, *p* = 0.742
RV mass index (g/m2)	*r* = 0.097, *p* = 0.075	*r* = 0.229, *p* < 0.001**	*r* = 0.004, *p* = 0.935
LV mass index (g/m2)	*r* = −0.027, *p* = 0.613	*r* = 0.072, *p* = 0.173	*r* = 0.041, *p* = 0.435
VMI	*r* = 0.117, *p* = 0.031*	*r* = 0.180, *p* = 0.001**	*r* = −0.010, *p* = 0.851

Legend: *WHO* world health organisation, *IPAH* idiopathic pulmonary arterial hypertension, *CTD* Connective tissue disease, *CHD* chronic heart disease, *CTEPH* chronic thromboembolic pulmonary hypertension, *RVEDV* right ventricular end diastolic volume, *RVESV* right ventricular end systolic volume, *RVSV* right ventricular stroke volume, *RVEF* right ventricular ejection fraction, *VMI* ventricular mass index, *LVEDV* left ventricular end diastolic volume, *LVESV* left ventricular end systolic volume, *LVSV* left ventricular stroke volume, *LVEF* left ventricular ejection fraction, *VMI* ventricular mass index

*P* values indicate comparisons between patients with PH and the other two groups. * signifies a *p* value < 0.05, ** signifies a *p* value < 0.025

A multivariate forward linear regression showed that RVESVI and systolic septal angle were significant independent predictors of RV insertion point T1 in patients with PAH.

In patients with left heart disease the RV insertion point T1, septal T1 and LV free wall T1 all showed significant correlations with cine CMR metrics of RV function. ΔT1_RS_ correlated significantly with diastolic and systolic septal angle in patients with left heart disease, (see Table 4).

**Table 4:** Linear regression analysis for RV insertion point T1

	Unstandardized Coefficients		Standardized Coefficients	t	*p*-value
	B	Std. Error	Beta		
Constant	.845	.073		11.609	< 0.001
RVESVI	.000623	0.000247	.209	2.524	0.008
RVEF	−0.000213	0.000500	−.034	−.425	0.921
VMI	.001	.001	.119	1.483	0.837
Septal angle systolic	.001	.000	.178	2.918	0.004
Relative Area Change	−.001	.001	−.066	−.755	0.655

Legend: *RVESVI* right ventricular end systolic index, *RVEF* right ventricular ejection fraction, *VMI* ventricular mass index

A total of 228/369 patients with PAH, 30/39 patients with PAH due to left heart disease and 29/82 patients without PAH also had right heart catheter within 1 month of CMR scanning (mean of 0 ± 1 days, range 15 days). Mean PAP for each patient group was as follows: patients with PAH: 44 ± 13; patients with PAH and left heart disease: 40 ± 12; patients without PAH 19 ± 3. Right heart catheter data is presented for patient subjects in Additional file 1**:** Table S3.

The mean RV insertion point T1 correlated significantly with the functional parameters mean PAP, mean right atrial pressure, and CI but not PVR, see Table 5. There was also a significant correlation with mixed venous oxygen saturation (SvO2). LV free wall T1 correlated significantly with CI and SvO2, whilst septal T1 did not correlate with any of the RHC parameters.

**Table 5:** Correlations between RHC parameters in septal, RV insertion point and LV free wall T1

RHC data	Septal T1 (ms)	RV insertion point T1 (ms)	LV free wall T1 (ms)
Mean RAP (mmHg)	*r* = 0.074, *p* = 0.256	*r* = 0.160, p = 0.011**	*r* = 0.025, *p* = 0.706
Mean PAP(mmHg)	*r* = 0.076, *p* = 0.248	*r* = 0.165, p = 0.011**	*r* = 0.025, p = 0.706
PCWP (mmHg)	*r* = −0.002, *p* = 0.982	*r* = −0.053, *p* = 0.434	*r* = − 0.032, *r* = 0.634
PVR (dyne/s/cm^3^)	*r* = 0.039, *p* = 0.567	*r* = 0.099, *p* = 0.145	*r* = 0.096, *p* = 0.160
CI (I/min/m^2^)	*r* = −0.003, *p* = 0.964	*r* = −0.079, *p* = 0.234	*r* = − 0.166.*p* = 0.012**
SvO2 (%)	*r* = −0.004, *p* = 0.956	*r* = − 0.152, *p* = 0.022**	*r* = − 0.168, *p* = 0.011**
SaO2 (%)	*r* = 0.119, *p* = 0.070	*r* = −0.081, *p* = 0.222	*r* = − 0.065, *p* = 0.322

Legend: *RAP* right arterial pressure, *PAP* pulmonary artery pressure, *PCWP* pulmonary capillary wedge pressure, *PVR* pulmonary vascular resistance, *CI* cardiac index, *SvO2* venous oxygen saturation, *SaO2* arterial oxygen saturation

*P* values indicate comparisons between patients with PH and the other two groups. * signifies a *p* value < 0.05, ** signifies a *p* value < 0.025

Patients with left heart disease showed significant correlations between RV insertion point T1 and mean PAP, mean right atrial pressure, and SvO2. Patients with left heart disease showed significant correlations with septal T1 and mPAP as well as SvO2. There were no correlations between LV free wall and right heart catheter parameters.

### Prognostic value of myocardial T1 in PAH

Fifty-nine patients with PAH died during the follow up period of 29 months (standard deviation: 8 months), of which 43 had PAH.

**Fig. 5:**
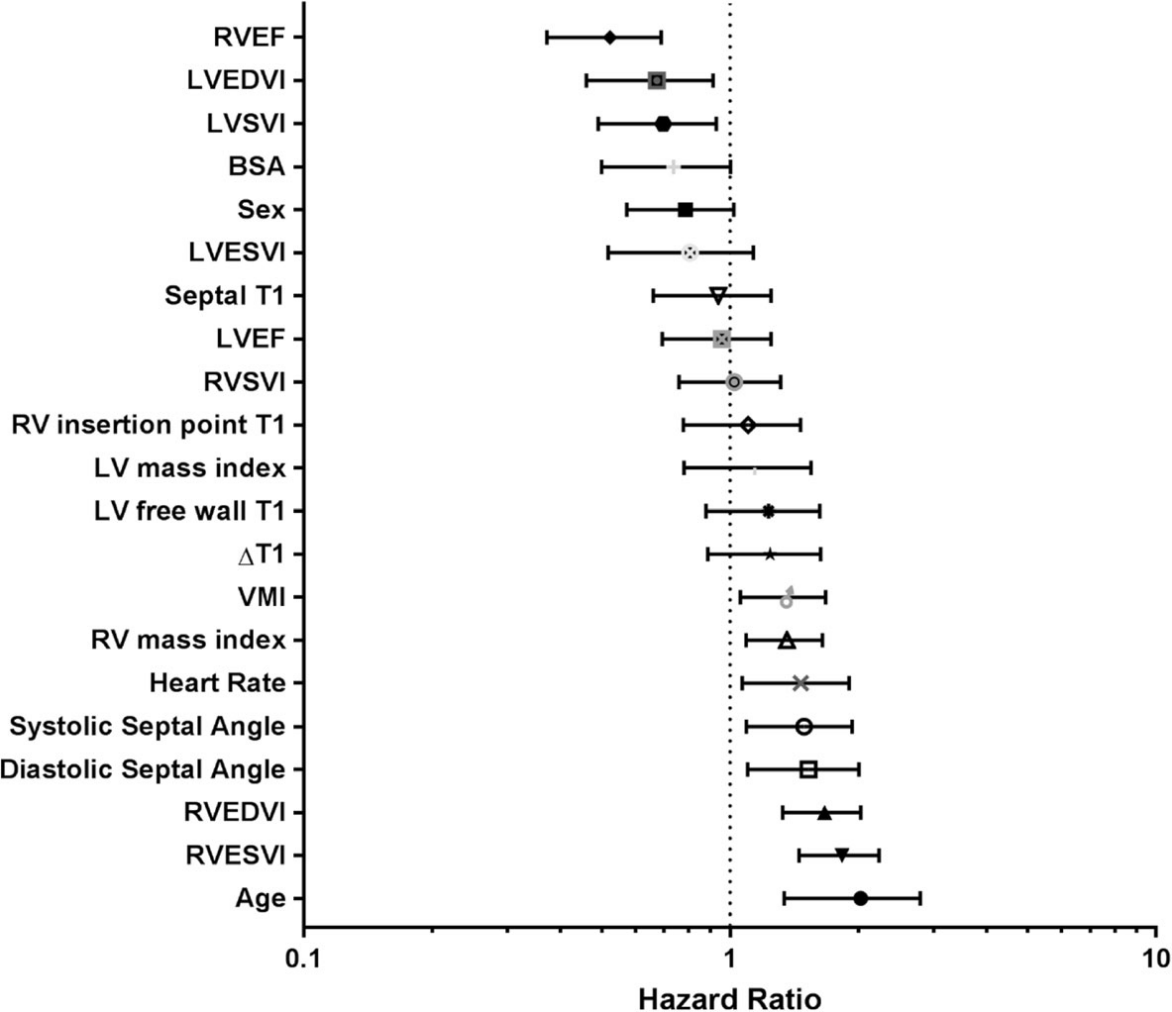
Forest Plot of hazard ratios for patient demographics and CMR derived metrics. RVEDV: right ventricular end diastolic volume. RVESV: right ventricular end systolic volume. RVSV: right ventricular stroke volume. RVEF: right ventricular ejection fraction. VMI: ventricular mass index. LVEDV: left ventricular end diastolic volume. LVESV: left ventricular end systolic volume. LVSV: left ventricular stroke volume. LVEF: left ventricular ejection fraction. VMI: ventricular mass index

Comparison of scaled Cox proportional hazard ratios are illustrated in Fig. 5. Univariate Cox proportional hazards regression analysis of patients with PAH showed that, septal, LV free wall and RV insertion point T1 were *not* associated with mortality (septal: *p* = 0.522; LV free wall: *p* = 0.258; RV insertion point: *p* = 0.688), see Table 6.

**Table 6:** Cox linear regression analysis for RV insertion point T1 in PAH. All variables have been normalised

	Univariate Hazard Ratio	Univariate 95% confidence interval	Univariate P Value
Age	1.938	1.342–2.798	< 0.001**
Sex	0.765	0.573–1.021	0.077
Septal T1	0.909	0.661–1.249	0.552
RV insertion point T1	1.067	0.778–1.464	0.688
LV free wall T1	1.195	0.879–1.624	0.258
ΔT1_RS_	1.205	0.888–1.634	0.237
RVEDVI	1.642	1.330–2.027	< 0.001**
RVESVI	1.804	1.453–2.240	< 0.001**
RVEF	0.506	0.372–0.690	< 0.001**
Systolic septal angle	1.454	1.093–1.934	0.011**
Diastolic septal angle	1.485	1.099–2.008	0.011**
RV mass Index	1.341	1.091–1.649	0.018**

Legend: *RVEDVI* right ventricular end diastolic volume index, *RVESVI* right ventricular end systolic volume index, *RVEF* right ventricular ejection fraction

### Reproducibility

T1 values in the septum, RV insertion point and LV free wall were tested for absolute agreement with a random two-way model. The intraclass correlation coefficients for the interventricular septum, RV insertion points and LV free wall T1 values respectively were: 0.841, 0.823 and 0.792 respectively. The interclass correlation coefficients for the septum, RV insertion point and LV free wall T1 values respectively were: 0.844, 0.742 and 0.618 respectively. Reproducibility was found to be excellent in all regions in healthy subjects, intraclass correlation coefficients for the interventricular septum, RV insertion points and LV free wall T1 values were: 0.898, 0.934 and 0.852 respectively. The interclass correlation coefficients for the septum, RV insertion point and LV free wall T1 values were: 0.972, 0.942 and 0.824 respectively.

## Discussion

Despite the clinical heterogeneity found in subtypes of PAH in the patients in this study, there were no significant differences found in myocardial T1 between the subtypes of this population. In particular, patients with left heart disease who were considered separately (due to a different aetiology and cardiac phenotype) were found to have no significant differences from patients with other forms of PAH, this may be because the causal mechanism of T1 differences in PAH be present to some extent in all PAH subtypes. However, this may also be due to multiple casual mechanisms causing elevated T1 in PAHdue to the heterogeneity of the disease.

Patients with CTD have been previously found to have elevated T1 when compared to controls [22], however we did not find that patients with CTD had significantly different T1 in any region when compared with patients with idiopathic PAH. This indicates that any tissue changes caused by the presence of cardiac connective tissue were not detectable in this study, likely having a less significant effect than those of PAH itself, or patients with connective tissue disease did not have significant LV disease.

Two control groups were used to evaluate changes in the myocardium in patients with PAH: age matched healthy subjects and patients without PAH. Patients without PAH had significantly higher RV insertion point T1 when compared to healthy subjects. Although these patients do not meet the criteria for PAH diagnosis, they have a mean PAP of 19.4 ± 2.7, which is higher than seen in a healthy subject population. Additionally, T1 changes are not specific to PAH, and therefore raised T1 in this group may be due to other disease such as connective tissue disease or left heart disease in this patient population.

Though both RV insertion point T1 and ΔT1_RS_ were successful at differentiating healthy subjects from patients with PAH, they were not successful at differentiating control patients from patients with PAH. ΔT1_RS_ was not significantly different between control groups, which suggests regional elevation of the insertion point T1 is characteristic of PAH rather than elevation of the whole myocardial T1. The lack of specificity of RV insertion point T1 indicates that processes responsible for the elevated RV insertion point T1 are not solely confined to patients with PAH.

The RV insertion points were the myocardial regions with the highest T1 and they correlated significantly with loss of RV function and RV remodelling. Systolic septal angle and RVESVI were independent predictors of RV insertion point T1, however these variables only predicted 12.4% of the variance seen in RV insertion point T1 in patients with PAH. This may indicate that mechanisms which are not explicable solely through cardiac volume, mass and pressure changes are responsible for the tissue changes represented by T1.

Animal models of PAH with increased native T1 and LGE present in the RV insertion points have been shown to have increased interstitial collagen associated with fibre disarray, and increased connective tissue density [11]. It has been shown in two patients with PAH that fibrosis was present in the RV insertion point and it has been suggested that this is the mechanism driving LGE in PAH [23]. However, those patients also had an increase in interstitial space and a small increase of fat. A CMR case study of one patient with idiopoathic PAH showed myocardial disarray with increased collagen and fat between fibre bundles in the myocardium (plexiform fibrosis). That study suggests that the cause of LGE is not pathological, and instead arises from an exaggeration of the myocardial disarray and plexiform fibrosis where fibres from the RV and LV cross. It is suggested this exaggeration of myocardial disarray may arise from a combination of RV hypertrophy and increased shear forces upon the interventricular septum [24].

Interventricular septal angle is a measure of deviation of the LV septum due to the high pressure in the RV, which correlated strongly with RV insertion point T1 in patients with PAH and was found to be an independent linear predictor of RV insertion point T1. Even in patients with left heart disease, who do not exhibit divergence of the LV septum to the same degree as other patients with PAH, ΔT1_RS_ correlated significantly with septal angle. The T1 value of the LV free wall, which would not experience stress/strain due to elevated ventricular pressure, was found to show no correlation between T1 and septal angle and was not elevated in patients with PAH when compared to patients without PAH. Therefore, our data suggests that the regional elevated native T1 in the insertion points is significantly related to the displacement of the interventricular septum in PAH. This supports the suggestions from histological studies that changes in myocardial T1 and the presence of LGE may be due to exaggerated fibre disarray in the RV insertion points of the myocardium due to stress on the septum and RV insertion points in PAH.

RV insertion point T1 was not an independent significant predictor of mortality in patients with PAH. Although LGE and elevated native T1 has been observed in the interventricular septum in PAH [3–5], elevated native T1 was not observed in this study. This may be because regions of interest were placed in the middle of the interventricular septum, whereas LGE imaging shows myocardial changes extending from the insertion points and therefore weakest enhancement at the central region of the septum.

Reproducibility data was found to be in line with that previously observed in patient T1 mapping studies [24]. The RV insertion points were found to be less reproducible than the interventricular septum. This may be due to higher T1 heterogeneity in this region leading to greater T1 variation when region of interest placement differs. T1 values tended to be less reproducible in patients with PAH than healthy subjects, which is consistent with other studies findings [24]. It may be that relatively poor breath-holding of the patient cohort contributes a greater degree of motion artefact resulting in weaker reproducibility.

Myocardial T1 did not provide additive diagnostic or prognostic information in this PAH cohort. It may be that myocardial changes are a secondary effect of cardiac remodelling and are therefore less relevant in this disease. However, it may also be that traditional region of interest based analysis of T1 is less suitable for this patient population due to heterogeneous T1 across the myocardium resulting in weaker reproducibility.

In conclusion, the RV insertion points have been shown to be the region where T1 changes are most significantly related to cardiac function in PAH. Myocardial T1 did not differ significantly between PAH subtypes including LHD. The T1 in the RV insertion point was significantly associated with RV volume and function in the patients scanned. Interventricular septal angle independently predicted RV insertion point T1 in patients with PAH suggesting that regional elevation in the RV insertion point may be linked to increased stress on the interventricular septal wall. T1 in patients with PAH was not found to be prognostic independent of RV size and function. Overall, T1 mapping does not appear to predict the subtype of PAH, or provide additive value over RV volume, mass and geometry in the prognostic evaluation of patients with PAH.

### Limitations

It is a limitation of this study that concurrent right heart catheter and CMR data were not present for all subjects. The follow up period for this study was relatively short, with 85% of patients alive at the follow-up date. Therefore, census at a later point may be required to provide more definitive evidence of whether RV insertion point T1 has independent prognostic value. Further work to evaluate regions of interest at the inferior and superior portions of the interventricular septum may clarify whether septal changes are present in these regions. The relationship between T1 and LGE in the RV insertion points was not evaluated here and further work may be warranted to evaluate this thoroughly in patients with PAH.

## Additional file

Additional file 1 **Table S1.** Subject characteristics for pulmonary artery sub groups. **Table S2.** Pulmonary artery hypertension subject. **Table S3**. Right heart catheterization characteristics of subjects. (DOCX 25 kb)

## Abbreviations

BSA: Body surface area
bSSFP: Balanced steady state free precession
CHD: Congenital heart diseases
CI: Cardiac index
CMR: Cardiovascular magnetic resonance
CTD: Connective tissue disease
CTEPH: Chronic thromboembolic pulmonary hypertension
ECG: Electrocardiogram
IPAH: Idiopathic pulmonary artery hypertension
LGE: Late gadolinium enhancement
LV: Left ventricle/left ventricular
LVEDV: Left ventricular end-diastolic volume
LVEF: Left ventricular ejection fraction
LVESV: Left ventricular end-systolic volume
MOLLI: Modified Look-Locker inversion recovery
PAH: Pulmonary artery hypertension
PAP: Pulmonary artery pressure
PCWP: Pulmonary capillary wedge pressure
PVR: Pulmonary vascular resistance
PVRI: Pulmonary vascular resistance index
RAP: Right atrial pressure
RV: Right ventricle/right ventricular
RVEDV: Right ventric ular end-diastolic volume
RVEF: Right ventricular ejection fraction
RVESV: Right ventricular end-systolic volume
SvO2: Mixed venous oxygen saturation
VMI: Ventricular mass index
