# Supplementary material for: Diagnostic and prognostic significance of cardiovascular magnetic resonance native myocardial T1 mapping in patients with pulmonary hypertension
Source: J Cardiovasc Magn Reson. 2018 Dec 3;20:78. doi: 10.1186/s12968-018-0501-8 (PMC6276188; doi:10.1186/s12968-018-0501-8)
Supplement: Supplementary file 1 — Table S1. Subject characteristics for pulmonary artery sub groups. Table S2. Pulmonary artery hypertension subject. Table S3. Right heart catheterization characteristics of subjects. (DOCX 25 kb) [file 12968_2018_501_MOESM1_ESM.docx]

Additional file 1

Table S1: Subject characteristics for PH sub groups

|  | PAH | LHD | PAH with LHD co-morbidity | PH-Lung | PAH with lung co-morbidity | CTEPH | PH-misc |
| --- | --- | --- | --- | --- | --- | --- | --- |
| **N** | 223 | 35 | 4 | 13 | 28 | 92 | 10 |
| **Demographics and PAH therapy** |  |  |  |  |  |  |  |
| Age (years) | 54.6±15.6 | 69.2±9.1 | 55.3±18.6 | 63.8±9.7 | 69.8±10.1 | 62.6±12.8 | 61.8±7.2 |
| Sex (%F) | 67.1% | 60% | 25% | 54% | 37% | 51.6% | 60.0% |
| *PAH therapy* |  |  |  |  |  |  |  |
| Monotherapy n (%) | N=56 (25%) | N=1 (3%) | N=2(50%) | N=2 (15%) | N=10(36%) | N=50 (54%) | N=1 (10%) |
| Combination | N=111 (50%) | N=0 (0%) | N=1(25%) | 1 N=1(8%) | N=18(64%) | N=16 (17%) | N=7 (70%) |
| Prostanoid | N=56 (25%) | N=0 (0%) | N=1(25%) | N=1 (8%) | N=0(0%) | N=9 (1%) | N=0 (0%) |
| On therapy at MRI | N=125 (56%) | N= 1(3%) | N=4(100%) | N=0(0%) | N=9(31%) | N=51 (55%) | N=5 (50%) |
| **Cardiac MRI** |  |  |  |  |  |  |  |
| RV insertion T1 (ms) | 1059±92 | 1078±10 | 1130±63 | 1058±86 | 1073±97 | 1055±84 | 1102±116 |
| Septal T1 (ms) | 981±70.0 | 981±70 | 1020±89 | 996.3±64 | 962±66 | 965±61 | 968±62 |
| LV free wall T1 (ms) | 963.9±67.1 | 982±76 | 958±91 | 992±61 | 955±56 | 964±72 | 983±31 |
| RVEDV Index (ml/m^2^) | 88.2±33.6 | 78.8±31.1 | 111.4±31.4 | 81.6±22.9 | 98.9±41.4 | 79.6±30.2 | 98.2±39.6 |
| RVESV Index (ml/m^2^) | 50.9±27.8 | 43.2±31.1 | 62.1±9.3 | 43.4±18.7 | 65.7±39.0 | 47.8±25.8 | 66.0±37.8 |
| RVSV Index (%) | 37.4±15.1 | 35.5±12.0 | 49.3±35.1 | 38.1±7.2 | 33.2±11.9 | 60.7±14.8 | 66.0±37.8 |
| RVEF (%) | 44.5±13.7 | 47.4±11.3 | 40.2±20.5 | 48.5±13.8 | 37.2±13.5 | 42.4±13.0 | 35.9±13.3 |
| Systolic septal angle (°) | 145.0±14.8 | 145.9±15.5 | 191.8±5.9 | 154.8±15.4 | 166.9±15.7 | 161.0±20.0 | 170.0±27.4 |
| Diastolic septal angle (°) | 138.2±10.3 | 138.3±10.3 | 151.3±9.7 | 140.8±8.61 | 149.3±10.9 | 141.2±8.6 | 143.6±13.8 |
| RV mass index (g/m^2^) | 14.2±5.22 | 14.2±5.2 | 23.8±8.3 | 18.7±8.9 | 23.2±11.9 | 19.1±9.2 | 21.5±9.0 |
| **N (RHC)** | 162 | 21 | 3 | 1 | 22 | 81 | 8 |
| mPAP (mmHg) | 45.8±14.7 | 39.9±12.1 | 50.3±12.1 | 35.6±6.8 | 46.7±8.5 | 42.3±12.4 | 51.1±9.1 |
| PVR (dyne/s/cm^3^) | 664±445 | 306±363 | 713±397 | 372±138 | 686±381 | 540±323 | 699±228 |

*Patients with PH-RESP, n=2 were receiving PAH therapy as had features of PAH and mild lung disease

*Patients with PH-LHD, n=1 was on receiving PAH therapy, this patient had features of PAH but a PCWP of 17, thus was not included in the PAH cohort.

Table S2: Subject characteristics for PAH subgroups

Characteristics

|  | IPAH | PAH CTD | PAH CHD | PAH other |
| --- | --- | --- | --- | --- |
| **N** | 82 | 89 | 31 | 20 |
| **Demographics** |  |  |  |  |
| Age (±SD) | 50.1±15.7 | 62.4±11.8 | 65.1±70.1 | 43.4±10.2 |
| Sex (%F) | 71.1% | 84.3% | 71.0% | 68.4% |
| **Cardiac MRI** |  |  |  |  |
| RV insertion T1 (ms) | 1060±81 | 1050±92 | 1081±111 | 1062±102 |
| Septal T1 (ms) | 993.5±73 | 976±67 | 965±70 | 973±63 |
| LV free wall T1 (ms) | 973±66 | 972±67 | 935±65 | 937±58 |
| RVEDV Index (ml/m^2^) | 85.0±26.9 | 74.2±12.7 | 110±48 | 94.4±33.8 |
| RVESV Index (ml/m^2^) | 48.9±23.5 | 48.2±26.8 | 62±39 | 52.3±24.3 |
| RVSV Index (%) | 36.1±13.5 | 33.9±11.9 | 47.6±18.2 | 52.3±24.3 |
| RVEF (%) | 44.1±13.3 | 43.6±13.9 | 47.1±14.5 | 45.2±13.7 |
| Systolic septal angle (°) | 171.9±28.7 | 159.8±23.6 | 171.6±26.8 | 165.9±21.9 |
| Diastolic septal angle (°) | 146.4±13.2 | 141.4±10.7 | 143.6±12.2 | 141.6±6..5 |
| RV mass index (g/m^2^) | 24.7±15.8 | 18.4±10.7 | 29.8±15.2 | 22.6±10.7 |
| **N (RHC)** | 51 | 79 | 14 | 18 |
| Mean PAP (mmHg) | 52.5±15.5 | 41.0±12.9 | 47.9±15.3 | 46.6±12.9 |
| PVR (dyne/s/cm^3^) | 858±521 | 556±393 | 497±253 | 604±403 |

Table S3: RHC characteristics of subjects

| **RHC data** | **Patients: PH (n=237)** | **Patients: PH LHD (n=39)** | **P value** | **Patients: without PH (n=37)** | **P value** |
| --- | --- | --- | --- | --- | --- |
| **Mean RAP (mmHg)** | 10.31±5.50 | 14.95±5.46 | *p<0.001*** | 6.55±3.35 | *<0.001*** |
| **Mean PAP(mmHg)** | 44.36±13.49 | 40.23±11.96 | *p=0.471* | 19.4±2.7 | *<0.001*** |
| **PCWP (mmHg)** | 11.85±3.87 | 23.68±6.89 | *p<0.001*** | 10.9±3.8 | *0.150* |
| **PVR (dyne/s/cm^3^)** | 611±402 | 379±81 | *p=0.004*** | 183±97 | *<0.001*** |
| **CI (I/min/m^2^)** | 2.66±0.79 | 0.764±0.163 | *p=1.000* | 2.78±0.64 | *0.408* |
| **SvO2 (%)** | 64.81±8.69 | 62.41±7.90 | *p=0.648* | 70.4±5.0 | *<0.001*** |
| **SaO2 (%)** | 93.63±4.03 | 94.4±3.57 | *p=1.000* | 96.3±2.9 | *<0.001*** |
